# Efficacy and safety of decompressive craniectomy in acute ischemic stroke patients treated with intravenous thrombolysis

**DOI:** 10.1186/s12883-022-03014-4

**Published:** 2023-03-28

**Authors:** Elyar Sadeghi-Hokmabadi, Farhad Mirzaei, Mohammad Yazdchi, Javad Jalili, Yalda Sadeghpour, Behzad Nemati-Anari, Mehdi Farhoudi, Pouneh HamianRoumiani

**Affiliations:** 1 Neurosciences Research Center (NSRC), Department of Neurology, Imam-Reza hospital, Tabriz University of Medical Sciences, Tabriz, Iran; 2 Department of Neurosurgery, Imam-Reza hospital, Tabriz University of Medical Sciences, Tabriz, Iran; 3 Department of Neurology, Imam-Reza hospital, Tabriz University of Medical Sciences, Tabriz, Iran; 4 Interventional Radiology, Department of Radiology, Imam-Reza hospital, Tabriz University of Medical Sciences, Tabriz, Iran; 5 Neurosciences Research Center (NSRC), Department of Neurology, Imam-Reza hospital, Tabriz University of Medical Sciences, Tabriz, Iran; 6 Department of Neurology, Imam-Reza hospital, Ardebil University of Medical Sciences, Ardebil, Iran; 7 Neurosciences Research Center (NSRC), Department of Neurology, Imam-Reza hospital, Tabriz University of Medical Sciences, Tabriz, Iran; 8 Neurosciences Research Center (NSRC), Department of Neurology, Imam-Reza hospital, Tabriz University of Medical Sciences, Tabriz, Iran

**Keywords:** Decompressive craniectomy, Tissue plasminogen activator, Ischemic stroke, Modified Rankin scale

## Abstract

**Introduction:**

The optimal timing for decompressive hemicraniectomy (DHC) after intravenous thrombolysis (IVT) remains unclear. This study in patients with acute ischemic stroke treated with IVT aimed to assess the safety of DHC and patient outcome.

**Methods:**

Data was extracted from the Tabriz stroke registry from June 2011 up to September 2020. In all, 881 patients were treated with IVT. Among these, 23 patients underwent DH. Six patients were excluded due to symptomatic intracranial hemorrhage (parenchymal hematoma type 2 based on SITS-MOST definition) after IVT, but other types of bleeding after venous thrombolysis, including HI1, HI2, and PH1 were not excluded; so the remaining 17 patients were enrolled in the study. Functional Outcome was defined as the proportion of patients who achieved mRS score of 2–3 (moderate disability), 4–5 (severe disability), or 6 (mortality) at 90 days after stroke. mRSwas assess by trained neurologist at the hospital clinic with direct interview Safety outcome was assessed by comparison of two scans just prior to and after craniectomy. Any new hemorrhage or worsening of previous hemorrhage was reported. Parenchymal hematoma type 2, based on ECASS II definition, was considered as major surgical complication. This study was approved by the local ethics committee of the Tabriz University of Medical Sciences (Ethics Code: IR.TBZMED.REC.1398.420).

**Results:**

At the three-month mRS follow up, six patients (35%) had moderate and five (29%) had severe disability. The outcome of death was observed in six patients (35%).Nine of 15 patients (60%) underwent surgery in the first 48 hours after onset of symptoms. No patient over 60 years of age survived to the three-month follow up; 67% of those who were under60 years and underwent DH in the first 48 hours had favorable outcome. Hemorrhagic complication was seen in 64% of patients but none was major.

**Conclusion:**

Results of this study showed that the rate of major bleeding and outcome of acute ischemic stroke patients who underwent DHC after IVT is comparable with the reported data in the literature and intentionally waiting for the fibrinolytic effects of IVT to disappear may not outweigh the benefits of DHC. Although the findings of the study should be interpreted with caution and larger studies are needed to confirm the results.

**Supplementary Information:**

The online version contains supplementary material available at 10.1186/s12883-022-03014-4.

## Introduction

Tissue plasminogen activator is an approved treatment for acute ischemic stroke within 4.5 hours after onset of symptoms. Its effect on improvement of functional recovery and raised survival has been previously reported [1, 2]. In some cases of unsuccessful recanalization, infarction can be malignant and intractable edema occurs. With conservative treatment alone, severe disability or death occurs in up to 80% of these patients [3]. Decompressive hemicraniectomy (DHC) significantly reduces mortality [4–6]. In patients who are treated with intravenous thrombolysis (IVT), the possibility of bleeding during and after surgery arising from coagulation impairment is a serious concern that may limit performing surgery; data about the safety of DHC in these patients are scarce [7].In the current study we aimed to assess the safety of DHC in acute ischemic stroke patients who were treated with IVT and their functional outcome.

## Material and methods

### Patients

This retrospective study was performed on data extracted from the Tabriz stroke registry [8, 9]. This study was performed at Imam Reza Hospital, a tertiary referral university hospital in Tabriz, Iran. This hospital is the only center in the province which provides 24/7 neurological services for IVT. All patients were treated according to latest AHA/ASA guidelines for management of patients with acute ischemic stroke. None underwent endovascular procedure. The first case of IVT was done in June 2011; up to September 2020, 881 patients were treated with IVT. Among these, 23 patients underwent DHC. For the purpose of this study, six patients were excluded due to symptomatic intracranial hemorrhage (parenchymal hematoma type 2 based on SITS-MOST definition) after IVT. This was done for two reasons: first, toreduce heterogeneity to be able to compare results of this study with other published trials of DHC which all were done exclusively for ischemic patients; and, second, to more accurately be able to evaluate the effect of DHC in reducing intracranial pressure (ICP), since patients with PH2 have poor outcomes with or without DHC not only because of edema and increased ICP but also due to the hematoma itself. The remaining 17 patients were enrolled in the study. One patient underwent two DHC procedures within 3 days.

### Variables

Data were recorded for all patients prospectively for demographic aspects of patients, pre-existing medical conditions, involved cerebral hemisphere, timetable of workflow (symptoms onset, needle time, surgery time), baseline stroke severity as assessed by the National Institutes of Health Stroke Scale (NIHSS), and stroke etiology based on TOAST classification. New bleeding after decompression (including subdural hematoma, subarachnoid hemorrhage or intra parenchymal hemorrhage), worsening of pre-existing intracerebral hemorrhage (ICH), or other life-threatening infections such as meningitis were considered to be post-operative complications.

### Surgical technique

DHC consisted ofa standard unilateral fronto-parieto-temporal craniectomy with duroplasty. The Dura was widely opened in a stellate fashion to the extent of bone decompression, and duraplasty was performed.

### Clinical management and follow up

All patients had brain CT scans immediately before, 24–36 hours after IVT, and again 24–72 hours after DHC. Functional outcome at 3 months was assessed using the modified Rankin Scale (mRS). Outcome was defined as the proportion of patients who achieved an mRS score of 3 and less (moderate disability),4–5(severe disability),or6 (mortality) at 90 days after stroke.

### Imaging assessment

**Fig. 1:**
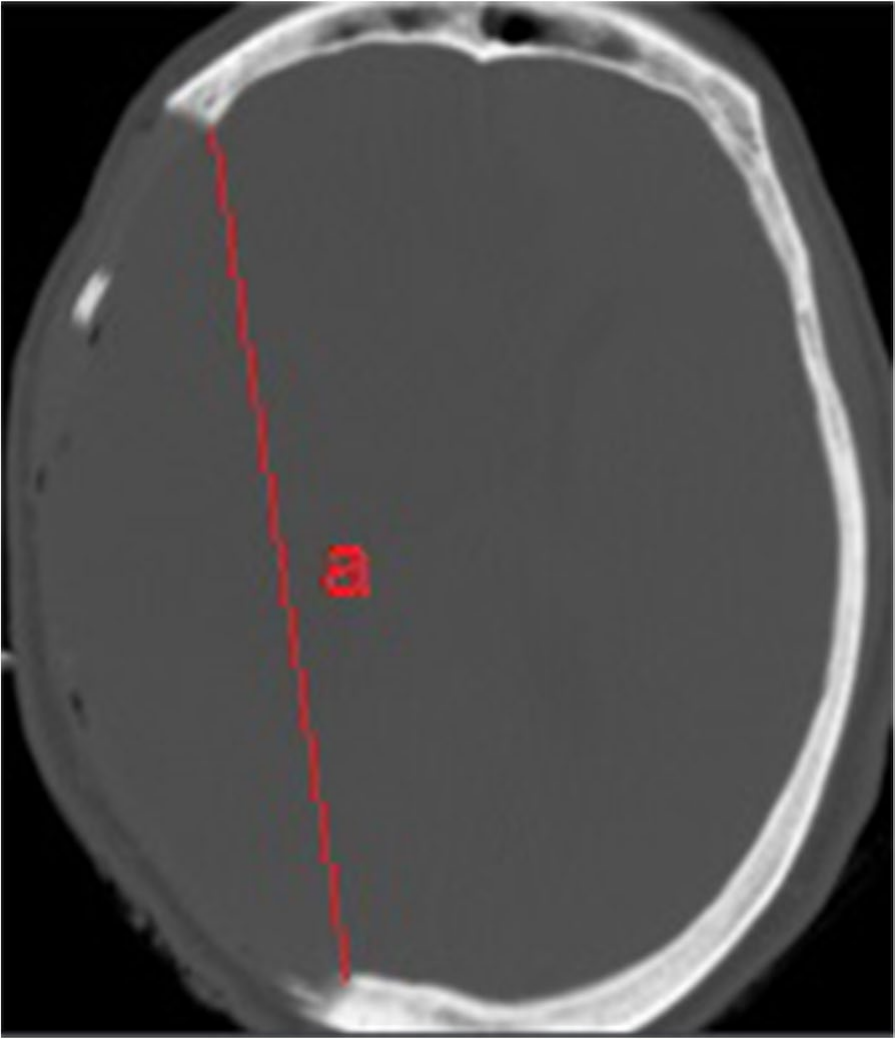
CT scan of a brain after craniectomy. a, diameter was measured by length of a line connecting inner tables of the cranium

Strokes were considered malignant if they involved more than 50% of the middle cerebral artery (MCA) territory (with or without anterior or posterior cerebral arteries territories) on CT or MR imaging with progressive neurological deficit and decrease in consciousness. All images before and after DHC were reviewed by two experienced neuroradiologists who were blinded to patient clinical outcome. Disagreement was resolved through consensus. Parenchymal hematoma type 2, base of ECASS II definition, was considered as a major surgical complication. Diameter of craniectomy was assessed by the length of a line connecting inner tables of the skull [10] (Fig. 1, supplement).

### Statistical analysis

Statistical analysis was done using SPSS 23. Variables with normal distribution are expressed as mean ± standard deviations and variables with non-normal distribution are presented as median and inter quartile range and Mann–Whitney U test was carried out. Statistical test results were considered significant at *p* < 0.05.

### Ethical considerations

DHC was performed with the written informed consent of the patient’s family. The present study was approved by the local ethics committee of the Tabriz University of Medical Sciences (Ethics Code: IR.TBZMED.REC.1398.420) informed consent was obtained from all patients or their legal guardian(s) and all methods were carried out in accordance with relevant guidelines and regulations.

## Results

Patients’ demographic and baseline characteristics and outcomes are summarized in Table 1. Median age was 53 and 70% were male. The median interval was 155 min between symptom onset and IVT, and 40 hours between symptom onset and surgery. DHC was performed within 48 hours after IVT in nine patients.

**Table 1:** Baseline characteristics of the patients (*n* = 17)

Baseline characteristics	Patients(n = 17)
Age,years, median(IQR)	53 (46–70)
Male, n (%)	12(70.6%)
Past medical history,	
Hypertension, n (%)	11(64.7%)
Diabetes mellitus, n (%)	4(23.5%)
Atrial fibrillation, n (%)	4(23.5%)
smoking, n (%)	1(5.9%)
Hyperlipidemia, n (%)	4(23.5%)
Previous history of stroke/TIA	1 (5.9%)
Previous history of MI/CABG/Angina	4 (23.5%)
Blood pressure on admission	
SBP, mmHg, median(IQR)	140(129–150)
DBP, mmHg, median(IQR)	85(80–100)
Blood sugar on admission,mg/dl (IQR)	142 (194–117)
Stroke etiology, n (%)	
Cardioembolic	5 (29.4%)
Probable	4 (23.5%)
Possible	1 (5.9%)
Large artery atherosclerosis	1 (5.9%)
Unknown	9 (52.9%)
Incomplete workup	7 (41.2%)
No finding on complete workup	2 (11.8%)
Others (Dissection)	2 (11.8%)
NIHSS on admission, median (IQR)	20 (22.5–16.5)
Symptom to needle time, minutes, median(IQR)	155(122–200)
Symptom to surgery, hours, median (IQR)	40(32–70)
LOS, days, median(IQR)	28(6–32.5)

At the three-month follow up, six patient (35%) had moderate disability (mRS of 2 or 3), five (29%) had severe disability (mRS of 4 or 5) and mortality (mRS 6) was observed in six (35%) (Table 2). Patients who were less than 60 years old had significantly better outcome than patients who were over 60 years old (moderate disability 60% vs. 0%, *p* < 0.01), but there was no significant difference between the time of surgery (within the first 48 hours after symptom onset vs. after 48 hours) and moderate disability (33% vs. 26%, *p* = 0.222).

**Table 2:** Three month outcomes of the patients (n = 17)

Outcome, n (%)	
Moderate disability (mRS 0–3)	6 (35.3%)
Severe disability (mRS 4–6)	11 (64.7%)
Mortality, n (%)	6 (35.3%)

CT scans before and after surgery was available for 11 patients. According to post-operative complications, new bleeding after surgery was seen in seven of 11 (64%) patients (hemorrhagic infarct type 2 in three and parenchymal hematoma type 1 and subdural hematoma in two patients each), but there was no major bleeding (parenchymal hematoma type 2) (Table 3).

**Table 3:** Imaging data (*n* = 11)

Infarct at right side, n (%)	6 (54.5%)
Infarct area	
MCA only, n (%)	8 (72.7%)
MCA plus PCA or ACA, n (%)	3 (27.3%)
Size of bone flap, Median (mm)	98.5 (106.25–83.75)
Post-operative bleeding	
Non major	7 (63.6%)
SDH HI2	2 (28.6%) 3 (42.9%)
PH1	2 (28.6%)
Major (PH2)	0 (0)

**Fig. 2:**
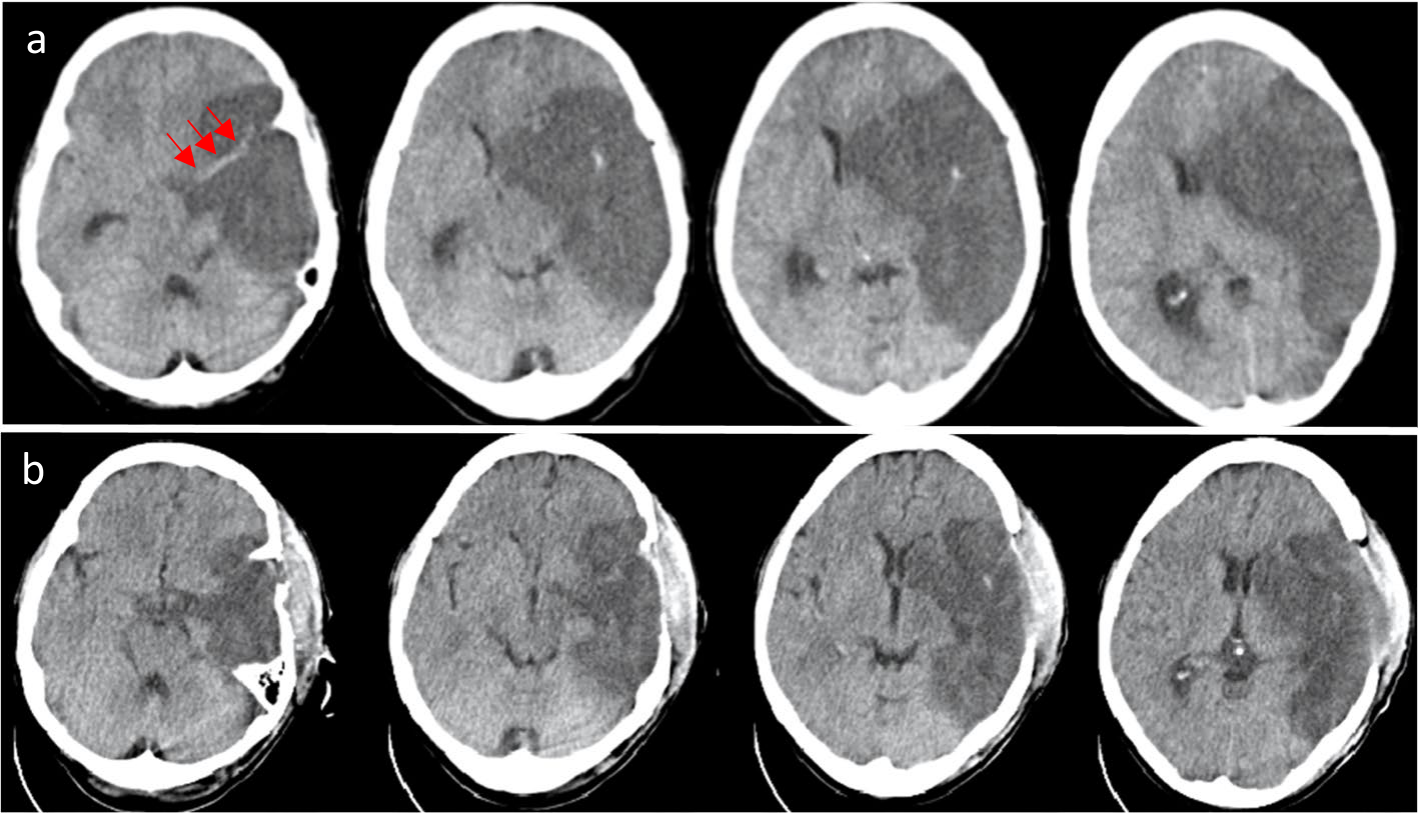
**a** Non-enhanced cranial computed tomography (NECCT) of a 44 gentleman with right side dense MCA sign (red arrows), mass effect and midline shift. **b** NECCT after surgery. Size of craniectomy is suboptimal. Surgery resulted in subdural hematoma but midline shift has resolved

Fig. 2 shows brain CT imaging of a patient before and after DHC. Surgery resulted in resolution of midline shift. Another patient was a 60 year old male. He underwent DHC 2 days after IVT, but the edema was progressive and the first surgery was not optimal, so a second surgery was done 3 days later. He did not develop postoperative hemorrhagic complications after either surgery. He had a mRS of 3 at the three-month follow up (Video 1and 2, supplement).

## Discussion

Increased favorable functional outcome and diminished mortality rate up to 20% after decompressive craniectomy has been reported in pooled analyses of randomized clinical trials [4, 5, 11, 12]. Reninke, in a recent systematic review and individual patient meta-analysis, found that the benefit of surgical decompression for space-occupying hemispheric infarction is consistent across a wide range of patients, with largest benefit seen in the first 2 days after stroke and in younger patients [11]. In patients who have been treated with IVT, the possibility of bleeding during and after surgery arising from coagulation impairment is a serious concern that may limit performing surgery. A number of studies have suggested that tissue plasminogen activator (tPA) exerts prolonged fibrinolytic effects which may continue for more than 24 h despite its short half-life (4–6 min) [7, 13]. The best and safest time point to perform DHC after IVT is unknown; performing early DHC may increase risk of hemorrhagic complication of surgery and performing late surgery may diminish its beneficial effects on outcome [11]. Data about safety of DHC in IVT patients are scarce. Takeuchi compared 20 patients who underwent DHC after IVTto20 patients who underwent DHC without IVT [13].He concluded that there were no significant differences in mortality and functional outcome between the two groups and DHC may be a safe and useful procedure in these patients. Fischer reported that DHC after intra-arterial (not intravenous) thrombolysis was safe and did not increase the rate of complications after DHC [7].

Regarding safety outcome, new bleeding complication was common after DHC (in 7 out of 11), but all were non major. Based on the results of our study, DHC after IVT in comparison with literature data in patients who underwent DHC and did not receive IVT, was not associated with higher rate of major bleeding, mortality, or additional neurologic disability, while the outcome was in the same range as those reported in the pooled analyses of RCTs [11, 12].

The novelty of this study was the tPA dose used for patients. Takeuchi used 0.6 mg/kg of tPA, the approved dose in Japan, but we used 0.9 mg/kg which is similar to the dose typically used in other countries, and the results showed that a higher dose (0.9 vs. 0.6 mg/kg) of tPA before DHC seems to be safe [13].

## Limitations

1. Systematic laboratory studies (such as a fibrinogen/fibrin-degradation products or the plasmin-2-antiplasmin complex) were not performed to assess specific fibrinolytic/ coagulation parameters before surgery. Therefore, we do not know whether fibrinolytic effects of IVT persisted at the time of the procedure. 2. This was a single center study with a limited number of patients; thus, optimal timing to perform DHC after IVT cannot be derived from our data. 3. Imaging data was not available for six patients due to data loss in a specific period of time and unrelated to patient outcome; however, the results must be interpreted with caution.

## Conclusion

The outcome of DHC after IVT was comparable with outcomes in other patients who underwent DHC and did not receive IVT. Although the rate of bleeding was high, none was major and DHC was safe even in the first 48 hours after symptom onset. Intentionally waiting for the fibrinolytic effects of IVT to disappear may not outweighs the benefits of DHC.

## Supplementary Information

**Additional file 1:** **Video 1.** Pateint’s condition after second surgery. Video 2. Patient’s functional outcome 3 month after surgery (mRS is 3).

## Data Availability

All data generated or analyzed during this study are included in this published article [and its supplementary information files].
